# Characterizing resting‐state networks in Parkinson’s disease: A multi‐aspect functional connectivity study

**DOI:** 10.1002/brb3.2101

**Published:** 2021-03-30

**Authors:** Mahdieh Ghasemi, Ali Foroutannia, Abbas Babajani‐Feremi

**Affiliations:** ^1^ Neural Engineering Laboratory Department of Biomedical Engineering University of Neyshabur Neyshabur Iran; ^2^ Department of Neurology Dell Medical School The University of Texas at Austin Austin TX USA; ^3^ Magnetoencephalography Lab Dell Children's Medical Center Austin TX USA

**Keywords:** dual regression, independent component analysis, Parkinson's disease, resting‐state network

## Abstract

**Purpose:**

Resting‐state functional magnetic resonance imaging (Rs‐fMRI) can be used to investigate the alteration of resting‐state brain networks (RSNs) in patients with Parkinson's disease (PD) when compared with healthy controls (HCs). The aim of this study was to identify the differences between individual RSNs and reveal the most important discriminatory characteristic of RSNs between the HCs and PDs.

**Methods:**

This study used Rs‐fMRI data of 23 patients with PD and 18 HCs. Group independent component analysis (ICA) was performed, and 23 components were extracted by spatially overlapping the components with a template RSN. The extracted components were used in the following three methods to compare RSNs of PD patients and HCs: (1) a subject‐specific score based on group RSNs and a dual‐regression approach (namely RSN scores); (2) voxel‐wise comparison of the RSNs in the PD patient and HC groups using a nonparametric permutation test; and (3) a hierarchical clustering analysis of RSNs in the PD patient and HC groups.

**Results:**

The results of RSN scores showed a significant decrease in connectivity in seven ICs in patients with PD compared with HCs, and this decrease was particularly striking on the lateral and medial posterior occipital cortices. The results of hierarchical clustering of the RSNs revealed that the cluster of the default mode network breaks down into the three other clusters in PD patients.

**Conclusion:**

We found various characteristics of the alteration of the RSNs in PD patients compared with HCs. Our results suggest that different characteristics of RSNs provide insights into the biological mechanism of PD.

## Graphical abstract

Deriving resting‐state networks using group independent component analysis. Applying an RSN score based on dual regression approach and statistical analysis. Identification of the significantly different regions of the cerebellum and occipital pole. The disintegration of the default mode network cluster into the other RSN clusters in PD.

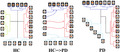

## 1 INTRODUCTION

Parkinson's disease (PD) is the second‐most common neurogenic disease in the world. It is accompanied by cognitive, emotional, and movement symptoms, such as tremor, slow motion, tightness, and movement and balance problems (Chaudhuri & Schapira, 2009). By the time of developing clinical symptoms, most of the dopaminergic cells in substantia nigra are lost (Hayashita‐Kinoh et al., 2006). Nonmotor symptoms such as emotional, cognitive, and behavioral deficits are seen in the stages of the disease. Emotional processing in Parkinson's patients compared with the healthy group has shown decreased neural communication in the bilateral putamen and increased neural communication in the right dorsomedial prefrontal cortex (Moonen et al., 2017). Cognitive and behavioral impairment occurs in 77% of people with PD, which causes changes in the neural connections of the cortical and subcortical structures such as the basal ganglia, thalamus, and frontal cortices (Mimura, 2007). For example, functional connections between the posterior and anterior hubs of the default mode network in Parkinson's patients affect the deterioration of cognitive functions (Weil et al., 2019). Moreover, an emotional task fMRI study demonstrated abnormal emotional valence in subcortical limbic structures in PD (Bell et al., 2019).

To explain some of the motor and nonmotor deficits found in patients with PD, the abnormal pattern of spontaneous activity and disrupted connectivity of the brain networks can be captured by fMRI at rest (Brooks & Pavese, 2011). Resting‐state fMRI (rs‐fMRI) connectivity analysis has great potential in investigating the underlying mechanism of PD. The rs‐fMRI reports the grid of brain areas that individually activated but functionally connected, known as resting‐state networks (RSNs). The RSNs reflect spontaneous neural activity signals between correlated temporal regions of the brain (Rektorova, 2014). There are mainly two large RSNs in PD: salience network (SN) and default mode network (DMN) (Bressler & Menon, 2010; Buckner et al., 2013; Raichle, 2015; Yuan et al., 2016). Previous studies have shown that alteration of the RSNs causes different emotional, cognitive, and behavioral impairment in neurodegenerative diseases (Lebedev et al., 2014) such as PD (Ghasemi & Foroutannia, 2019; Ghasemi et al., 2021; Wu et al., 2009) and other neurological and psychiatric diseases mainly Alzheimer's disease (Badhwar et al., 2017), dementia (Gratwicke et al., 2020), depression (Greicius et al., 2007), and schizophrenia (Whitfield‐Gabrieli et al., 2009). RSNs are usually identified by the seed‐based method (Biswal, 2012; Fernández‐Seara et al., 2015; Tahmasian et al., 2015) or a data‐driven method based on independent components analysis (ICA) (Beckmann et al., 2005; Erhardt et al., 2011). In the following, some related findings regarding RSN connection changes in PD will be mentioned.

Tessitore, Amboni, et al. (2012) found diminished functional connectivity (FC) of the right medial temporal lobe and the bilateral inferior parietal cortex within the DMN in PD patients compared with healthy controls (HCs) (Tessitore, Amboni, et al., 2012). Disbrow et al. (2014) assessed the DMN and executive RSNs in PD patients and HCs and found that neuropsychological investigations were not significantly different in the executive RSNs between two groups, although there was a reduced FC in the DMN of PD patients (Disbrow et al., 2014). Tahmasian et al. reported aberrant FC in bilateral inferior parietal lobule and the supramarginal gyrus in PD patients and found that these regions formed an interconnected network in PD patients, mainly with the DMN (Tahmasian et al., 2017). In another article, functional connections of the SN increase in group comparisons PD and HC (Navalpotro‐Gomez et al., 2020). Tessitore's et al. also examined the salience, central executive, and DMN and showed that there was an increase in connectivity in the SN and DMN, as well as reduced connectivity in the central executive networks (Tessitore et al., 2017).

Previous studies have shown changes in resting brain network connectivity in PD patients, and limited studies have examined the disruption of the main RSNs in PD. The main questions in this research are about the RSN changes from different points of view. First, what is the inter‐individual variability of the RSNs? Second: what is the most significant different region of the RSNs? And third, what areas do RSNs break down when they are disrupted and disintegrated in PD? To answer these questions, we compared the connectivity of within and between RSNs among PD patients and HCs to investigate alterations of the brain network in PD. We sought to address the interindividual variability of the brain network in PD by proposing a subject‐specific score—named an RSN score. After that, we investigated a voxel‐wise comparison between extracted RSNs by randomizing method and a hierarchical clustering analysis between the RSNs based on connectivity measures in the PD patient and HC groups.

## 2 METHODS

### 2.1 Overall procedure

**FIGURE 1:**
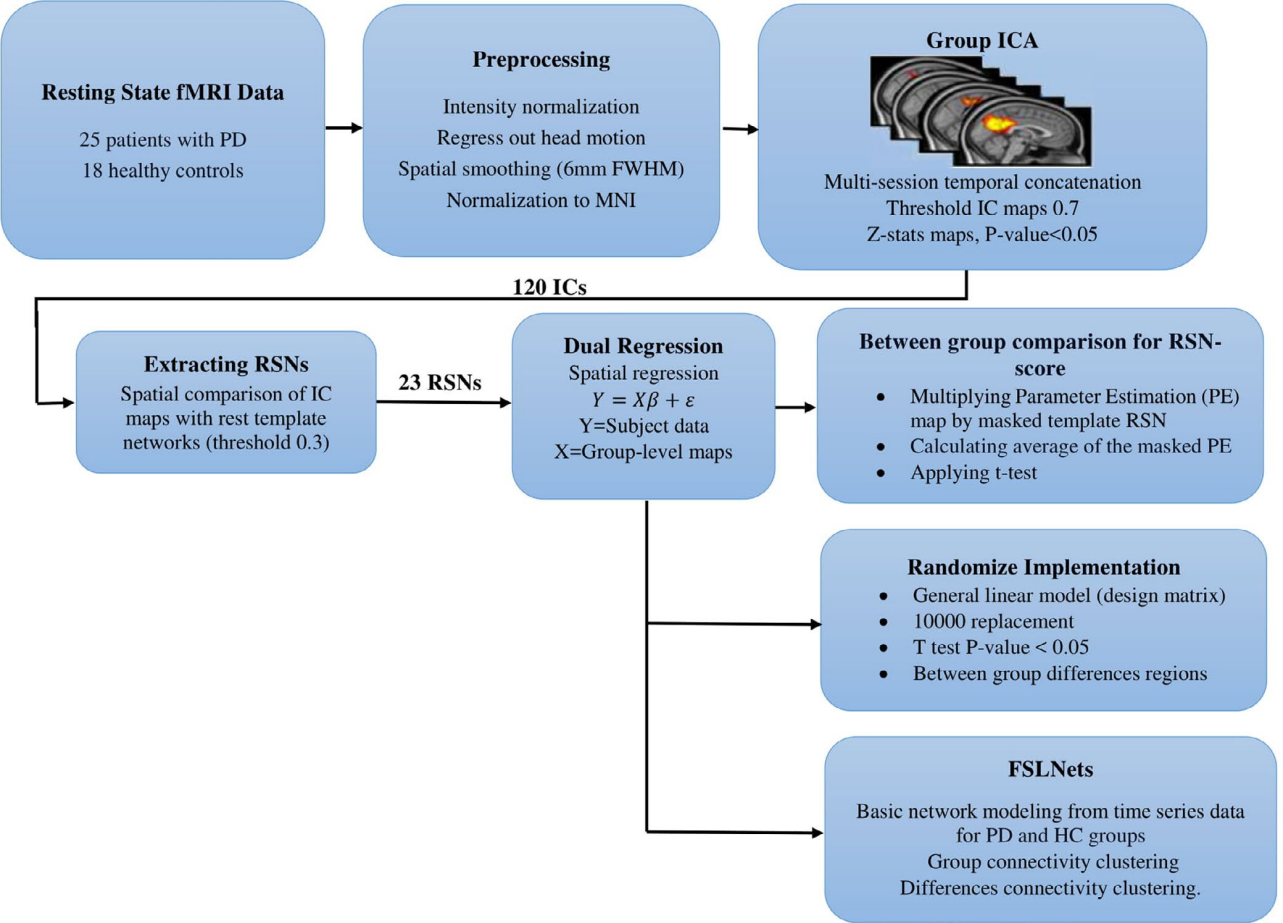
Overall procedures of this study

The overall procedures of this study are shown in Figure 1. After preprocessing the rs‐fMRI data, independent component (IC) group extraction and statistical tests were performed. Twenty‐three RSNs were separated from other noisy components by overlapping the spatial maps of ICs and resting‐state template reference networks. After applying the dual‐regression analysis and finding subject‐specific IC maps, an RSN score was calculated for each IC of all subjects. Then, the RSN scores of all components were statistically compared among the two groups using the nonparametric Kruskal–Wallis test. Next, a comparison of one‐by‐one individual RSN maps was made between the two groups via randomization. Basic network modeling was performed using the IC’s time series using FSLNets (http://fsl.fmrib.ox.ac.uk/fsl/fslwiki/FSLNets; RRID: SCR_002823) for PD and HC groups based on full and partial correlation measures. Finally, an additional structural MRI analysis was carried out with the FSL‐VBM tool to investigate voxel‐wise differences in the volume and topography of the gray matter in both the PD patient and HC groups.

### 2.2 Patients

Data for this study were randomly selected from the Parkinson's Progression Markers Initiative database (PPMI; RRID: SCR_006431) (Marek et al., 2011). The PPMI database that supports the findings of this study is openly available online (www.ppmi‐info.org/data), and up‐to‐date information on the PPMI study is online at www.ppmi‐info.org. PPMI is a public–private partnership funded by the Michael J. Fox Foundation for Parkinson's Research and other funding partners. (A listing of these partners can be found at www.ppmi‐info.org/fundingpartners.) We included 25 PD patients (14 males, 61.8 ± 10.4 years of age) and 18 age‐ and gender‐matched HCs (14 males, 64.2 ± 9.8 years of age) in this study (Table 1). Patients with PD were diagnosed on the basis of the United Kingdom PS Society Brain Bank criteria. The severity of Parkinson's disease was assessed with the Hoehn and Yahr scale. Healthy participants reported no history of previous neurological or psychiatric disorders and had no first‐degree relatives with idiopathic PD (parents, siblings, or children). This clinical study will be conducted according to the protocol and in compliance with Good Clinical Practice (GCP), with the Declaration of Helsinki1 (Version 2008), and with other applicable regulatory requirements [Taken from PPMI website, document title: “Clinical Study Protocol”].

**TABLE 1:** Demographic, clinical, and fMRI movement information in patients with PD and HCs

			PD	HC	*p*‐value
Number			25	18	–
Male/Female			14 / 9	14 / 4	.69
Hand Dominance (Right/Left)			25/0	18/0	–
Age (Mean + *SD*)			61.8 ± 10.4	64.2 ± 9.8	.41
Weight			79.76 ± 12.39	81.77 ± 11.68	.58
Hoehn and Yahr			1.84 ± 0.47	–	–
Movement Parameters	Translation (mm)	X	0.462 ± 4.023	0.043 ± 3.532	5.525e−07
		Y	−0.027 ± 0.732	0.171 ± 2.279	3.803e−04
		Z	0.237 ± 3.028	0.360 ± 3.385	7.7e−3
	Rotation (radian)	X	0.009 ± 0.124	0.019 ± 0.133	1.446e−04
		Y	0.005 ± 0.152	−0.010 ± 0.161	2.345e−06
		Z	−0.049 ± 0.110	−0.042 ± 0.181	1.17e−5

Abbreviations: HC, healthy controls; PD, Patients with Parkinson disease.

### 2.3 Neuroimaging protocol

Resting‐state blood oxygen level‐dependent (BOLD) data were collected on a 3‐T Siemens Trio Tim MRI scanner (Siemens, Munich, Germany) with 12‐channel scans from September 2013 to January 2014. (The acquisition parameters are detailed in the following link: https://www.acraccreditation.org/~/media/ACRAccreditation/Documents/MRI/Requirements.pdf) For each subject, a structural high‐resolution T1‐weighted MRI and a total of 205 functional T2‐weighted images (repetition time/echo time [TR/TE] =2400/25 ms; flip angle = 80º; slice thickness = 3.29 mm; field of view [FOV] =222 mm; matrix size of 68 × 66 matrix) were acquired.

### 2.4 Structural MRI analysis

In order to verify the typical pattern of atrophy in PD patients, we evaluated gray matter (GM) volume differences between patients with PD and HCs. The voxel‐based morphometric analysis was performed on structural MRI data using the FSL‐VBM toolbox (FSL, RRID: SCR_002823) (Ashburner & Friston, 2000). First, the skull‐stripped structural images of the brain were extracted, and the brain tissues were segmented (Zhang et al., 2001). The resulting gray matter volume images were normalized to the standard Montreal Neurological Institute (MNI) 152 using FNIRT. Voxel‐wise nonparametric statistical test (*n* = 10,000) was applied in FSL to identify significantly different (*p* < .05; family‐wise error [FWE]‐corrected) GM voxels among the two groups using the threshold‐free cluster enhancement (TFCE) technique (Smith & Nichols, 2009).

### 2.5 Preprocessing of RS‐FMRI data

All rs‐fMRI data were preprocessed using FSL (FSL5.0.6, http://www.fmrib.ox.ac.uk/fsl; RRID: SCR_002823) (Jenkinson et al., 2012), by applying the following procedures: spatial smoothing using a Gaussian kernel of FWHM 6 mm, head motion and slice‐timing corrections, intensity normalization, removing nonbrain tissue by BET in FSL, and spatial normalization to MNI‐152 standard space using nonlinear registration FNIRT in FSL (www.fmrib.ox.ac.uk/analysis/techrep). The first, five volumes were discarded to remove initial transient effects. Moreover, data were temporally high‐pass filtered at 0.01 Hz.

### 2.6 Group independent components analysis

We generated RSNs using FSL’s Melodic tool version 3.14 (Beckmann & Smith, 2004). The concatenated multiple fMRI datasets were decomposed using ICA to identify large‐scale patterns of functional connectivity in the subject population. The IC maps were thresholded using the false‐discovery rate at *p* < .05 (Beckmann & Smith, 2004). The dataset was decomposed into 120 components, including artifactual and desired components. For extracting RSNs, we spatially correlated all IC maps to a set of 20 reference resting‐state template networks (Laird et al., 2011) with threshold 0.3 using the “fslcc” tool in FSL. A list of brain regions associated with the resting‐state template networks is presented in Table 2. We name these 20 desired regions as extracted RSNs (interested ICs) hereafter.

**TABLE 2:** A list of brain regions associated with the resting‐state template networks used in this research

ICN number	Brain regions
1	Limbic and medial temporal areas
2	Subgenual ACC and OFC
3	Bilateral BG and thalamus
4	Bilateral anterior insula/frontal opercula and the anterior aspect of the body of the cingulate gyrus
5	Midbrain
6	Superior and middle frontal gyri
7	Middle frontal gyri and superior parietal lobules
8	Ventral precentral gyri, central sulci, postcentral gyri, superior and inferior cerebellum
9	Superior parietal lobule
10	Middle and inferior temporal gyri
11 & 12	Lateral and medial posterior occipital cortices
13	Medial prefrontal and posterior cingulate/precuneus areas
14	Cerebellum
15	Right‐lateralized frontoparietal regions
16	Transverse temporal gyri
17	Dorsal precentral gyri, central sulci, postcentral gyri, superior and inferior cerebellum
18	Left‐lateralized frontoparietal regions
19	Template mismatch errors in the brain map
20	Algorithmic abnormality occurring

Abbreviations: ACC, Anterior cingulate cortex; BG, Basal ganglia; ICN, Independent component network; OFC, Orbitofrontal cortex.

### 2.7 Group difference in rsn score

After extraction of the group‐specific RSNs by ICA, a dual‐regression approach was used to identify subject‐specific spatial maps and associated temporal dynamics for each subject. We defined “RSN score” for each individual IC as the mean value of spatial regression maps (parameter estimation) across all IC voxels. The RSN scores of all ICs were compared among the PD patient and HC groups using the nonparametric Kruskal–Wallis test to find ICs with significantly different scores (*p* < .05).

### 2.8 Voxel‐wise network analysis

A voxel‐wise statistical test using a randomized approach was performed to compare the subject‐specific maps of patients with PD and HCs for each IC (Nichols & Holmes, 2002). The general linear model (GLM) matrix, nonparametric permutation testing (10,000 permutations), and the “fslstats,” “fslmaths,” and cluster tools in FSL were used to find significant differences in the spatial maps of the PD patient and HC groups (Nickerson et al., 2017; Reineberg et al., 2015).

### 2.9 Network modeling

The time‐domain dependency of the RSNs makes a network model of connections. The strength of the connections between different regions leads to a hierarchical network. Brain network modeling based on hierarchical clustering reorders the ICs and brings together highly correlated components to form larger‐scale networks. Hierarchical clustering analysis was performed using the FSLNets tool for the PD patient and HC groups.

## 3 RESULTS

### 3.1 Structural analysis

The results of our structural MRI analysis using VBM‐FSL revealed no significant difference in the gray matter of PD patients and HCs (*p* > .33).

### 3.2 Group RSN analysis

**FIGURE 2:**
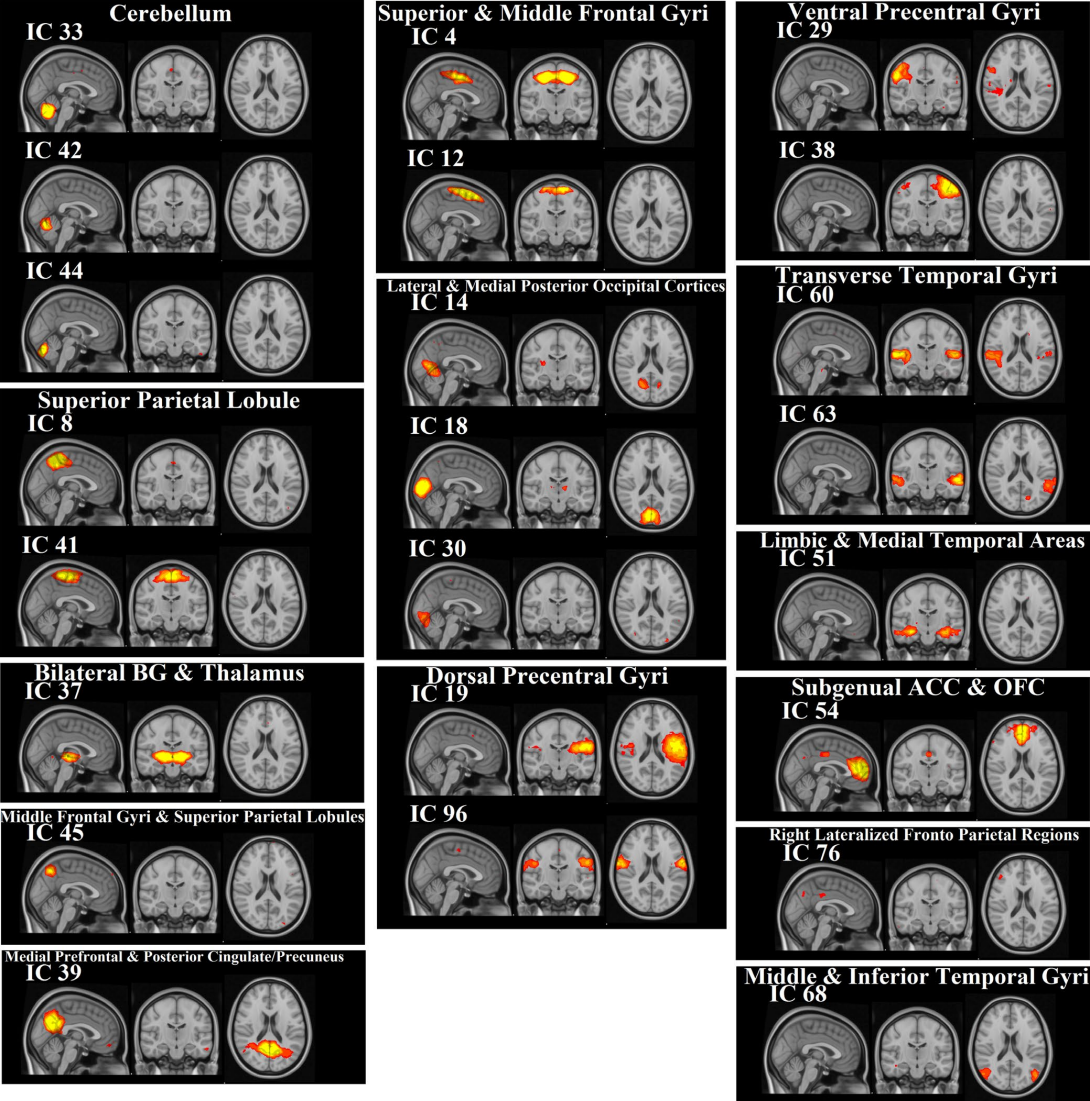
Group ICs results. The extracted 23 components were grouped into 14 categories based on spatial overlapping to a resting‐state template network: cerebellum (IC 33, 42, 44); superior parietal Lobule (IC 8, 41); Bilateral basal ganglia (BG) and thalamus (IC 37); middle frontal gyri and superior parietal lobules (IC 45); medial prefrontal and posterior cingulate/precuneus (IC 39); superior and middle frontal gyri (IC 4, 12); lateral and medial posterior occipital cortices (IC 14, 18, 30); dorsal precentral gyri (IC 19, 96); ventral precentral gyri (IC 29, 38); transverse temporal gyri (IC 60,63); limbic and medial temporal areas (IC 51); subgenual anterior cingulate cortex (ACC) and orbitofrontal cortex (OFC) (IC 54); right‐lateralized frontoparietal regions (IC 76); and middle and inferior temporal gyri (IC 68)

We identified 120 group ICs from 43 subjects. IC maps were thresholded at a level of 0.7 (threshold IC maps [signal > noise] = 0.7). By overlapping these 120 ICs with the rest network templates, 97 ICs were associated with artifacts, and 23 ICs were identified as brain components (Figure 2). It is noteworthy that some brain areas were associated with more than one IC (e.g., the superior and middle frontal gyri in IC 4 and 12, and the cerebellum in IC 33, 42, and 44). The anatomical locations of ICs were identified using the Harvard–Oxford cortical and subcortical atlases and are listed in Table 3.

**TABLE 3:** The regions of extracted group RSNs. The peak coordinates of maximum intensity, cluster size, the mean, and standard deviation of the extracted RSNs (ICs) are listed. The peak coordinates are given in MNI space, and anatomical information is represented according to Harvard–Oxford Atlas. The maximum value reflects the peak value of the IC map

IC number	Consistent RSN	Voxels	MAX			Mean	Standard deviation
			X	Y	Z		
4	Juxtapositional Lobule Cortex (formerly Supplementary Motor Cortex)	13,398	36	61	59	0.114	1.0722
8	Precuneous Cortex	9,694	44	41	65	0.077	0.8031
12	Juxtapositional Lobule Cortex (formerly Supplementary Motor Cortex)	8,526	45	65	67	0.067	0.7769
14	Lingual Gyrus	8,530	49	31	38	0.067	0.7413
18	Supracalcarine Cortex	7,451	48	19	39	0.063	0.7704
19	Central Opercular Cortex	10,734	70	55	48	0.069	0.6418
29	Postcentral Gyrus	9,355	19	48	59	0.059	0.5778
30	Lingual Gyrus	9,079	53	21	30	0.068	0.7428
33	Cerebellum	5,574	41	33	22	0.047	0.7209
37	Left Thalamus	7,304	51	53	35	0.053	0.6954
38	Precentral Gyrus	9,486	67	54	63	0.066	0.6492
39	Precuneous Cortex	9,234	43	34	49	0.069	0.7523
41	Precentral Gyrus	7,987	44	48	70	0.054	0.6093
42	Cerebellum	7,055	35	31	25	0.053	0.6597
44	Cerebellum	7,242	50	24	22	0.064	0.7788
45	Precuneous Cortex	7,131	34	32	60	0.048	0.5792
51	Right Hippocampus	4,565	28	53	28	0.047	0.5051
54	Paracingulate Gyrus	6,775	45	87	42	0.048	0.5805
60	Heschl's Gyrus (includes H1 and H2)	4,745	14	52	41	0.043	0.4989
63	Superior Temporal Gyrus, posterior division	5,539	77	41	39	0.049	0.5286
68	Lateral Occipital Cortex, inferior division	5,173	20	30	36	0.049	0.5647
76	Angular Gyrus	5,995	21	33	58	0.060	0.6460
96	Postcentral Gyrus	2,668	16	60	49	0.032	0.4228

### 3.3 RSN scores in PD and HC

**FIGURE 3:**
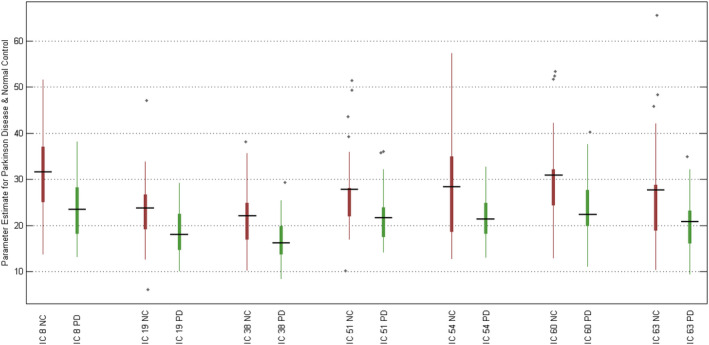
Boxplot of the RSN scores of 7 ICs that were significantly different among PD patients and HCs. Note that the RSN scores of patients with PD are significantly smaller than those in HCs, which indicates a reduction in brain connections in PD

We extracted subject‐specific RSN scores and examined the group differences as defined in Section 2.7. The RSN scores of 7 ICs in patients with PD were significantly smaller than that in HCs (*p* < .05). Statistical results and boxplot are shown in Table 4, and Figure 3, respectively. It is noteworthy that the average RSN scores of almost all ICs in patients with PD were smaller than that in HCs.

**TABLE 4:** The result of between‐group comparisons of RSN scores in which only the significantly different RSNs are listed (*p* < .05)

IC Number	*p*‐value	Region
IC 18	.0052	Lateral and medial posterior occipital cortices
IC 19	.0342	Dorsal precentral gyri, central sulci, postcentral gyri, superior and inferior cerebellum
IC 38	.0241	Ventral precentral gyri, central sulci, postcentral gyri, superior and inferior cerebellum
IC 51	.0463	Limbic and medial temporal areas
IC 54	.0291	Subgenual ACC and OFC
IC 60	.0222	Transverse temporal gyri
IC 38	.0422	Transverse temporal gyri

Abbreviations: ACC, Anterior cingulate cortex; OFC, Orbitofrontal cortex.

### 3.4 Voxel‐wise differences

**FIGURE 4:**
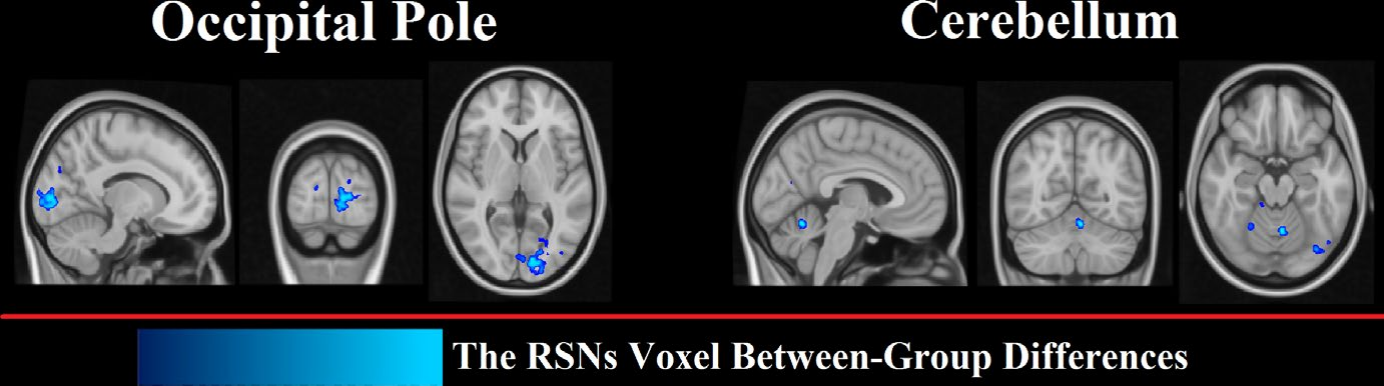
Voxel‐wise spatial regression analysis to compare subject‐specific maps of patients with PD and HCs. Voxels that were significantly different in the two groups are overlaid with a blue color on the MNI standard atlas

Based on methods described in Section 2.8, we found that individual differences between PD and HC were associated with some voxels in 2 regions: the occipital pole and cerebellum (Figure 4). The size and location of significant clusters identified by the dual‐regression analysis are listed in Table 5.

**TABLE 5:** The result of the voxel‐wise comparison of the subject‐specific spatial IC maps. Significant clusters identified by randomizing analysis are listed. Anatomical locations of the clusters, maximum, mean, and standard deviation of intensity, and coordinate (in MNI space) of the maximum intensity are listed

IC number	Anatomical cluster	Voxels	MAX intensity	MAX coordination			Mean	Standard deviation
				X	Y	Z		
68	Cerebellum	55	0.99	47	33	27	0.0002	0.0066
96	Occipital Pole	224	0.96	51	18	38	0.0037	0.0305

### 3.5 Connectivity clustering in two groups

**FIGURE 5:**
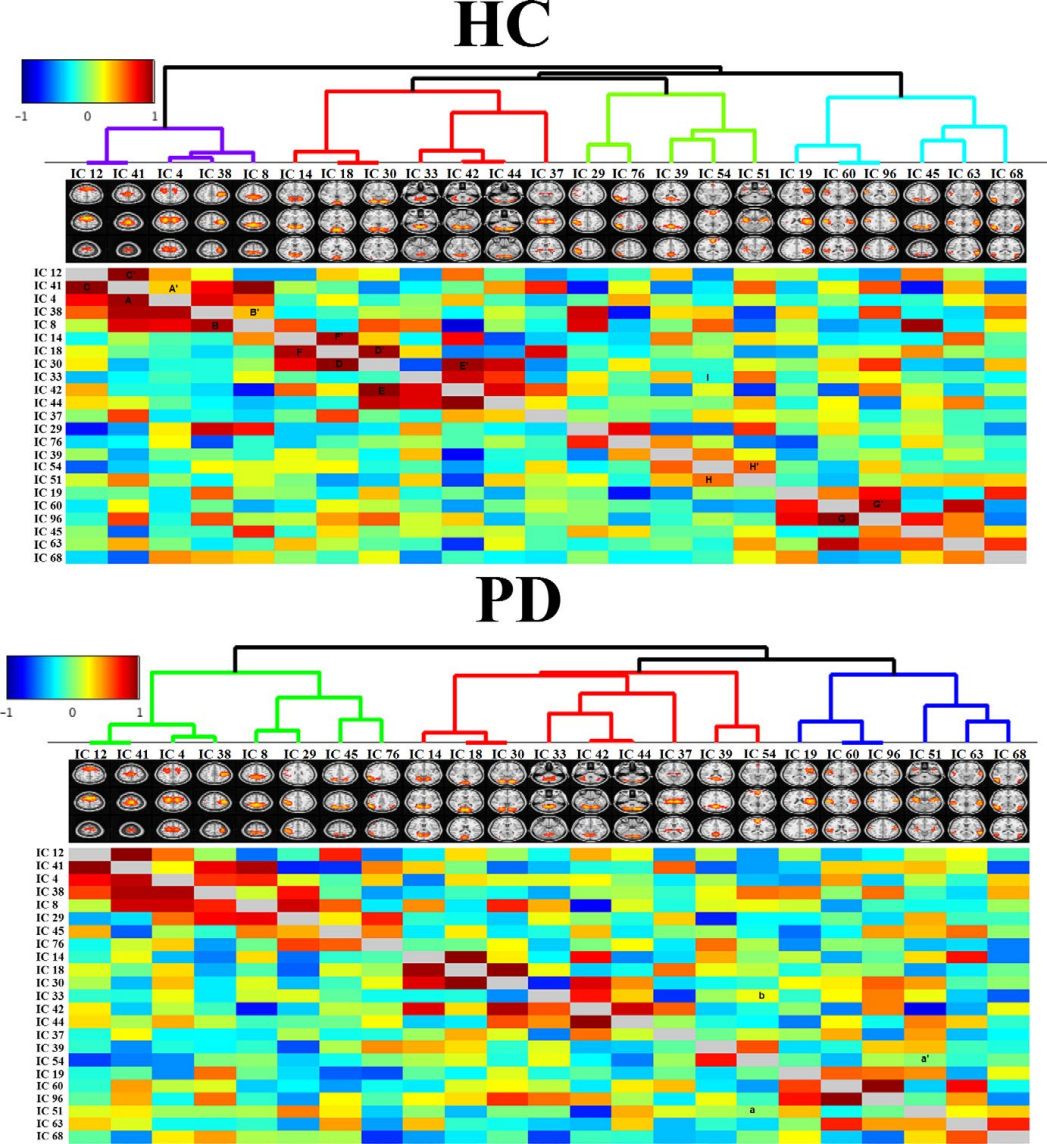
The correlation matrix was extracted from the time series associated with 23 RSN components in HCs (top panel) and patients with PD (bottom panel). Each row or column is a set of correlations between a network node (IC) and all other nodes (ICs). The lower and upper triangular matrices in each panel represent the full and partial correlations, respectively. According to the strength of correlations, the matrix of the nodes was arranged and hierarchical clustering was made

We compiled the matrix of full correlation and partial correlation of RSN time courses derived by each group separately. The network grid of HC and PD groups is shown in Figure 5. The nodes (RSNs in this case) clustered hierarchically according to functional connectivity. Dendrogram coloring indicated main clusters with high temporal correlations. There are four main clusters of RSNs with more similarity in HC.

The lower and upper triangular matrices represent the full and partial correlations, respectively. In the HC group (top panel of Figure 5), strong full correlations can be seen between IC 4 and IC 41—marked as A in Figure 5. On the other hand, the partial correlation between them was not very strong—marked A′ in Figure 5—indicating indirect functional connectivity between these nodes. This is true for IC 8 and IC 38, marked as B and B′ in Figure 5. The symmetric full and partial correlation connections were observed between IC 41 and IC 12—marked as C and C′ in Figure 5—that indicates a strong direct functional connectivity between these two nodes. Symmetric connections were seen between IC 30 and IC 18 (marked as D and D′), IC 30 and IC 42 (marked as E and E′), and IC 60 and IC 96 (marked as F and F′).

**FIGURE 6:**
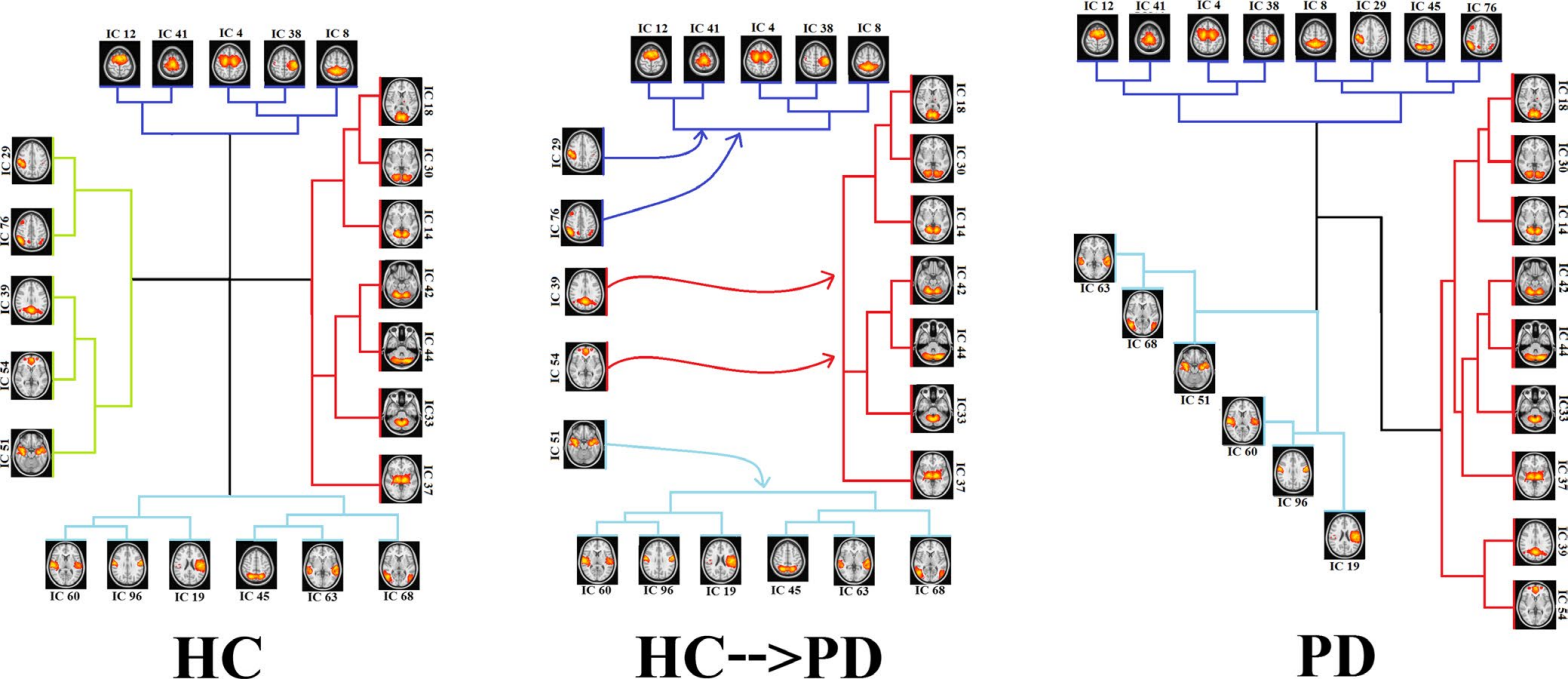
Clusters of ICs in patients with PD and HCs. Note that 4 clusters in HCs were reduced to 3 clusters in patients with PD. The cluster with green color in HCs contains IC 29, 76, 39, 54, and 51 and is related to the DMN. This cluster no longer exists as a separate cluster in patients with PD

Some strong connections in patients with PD were the same as those in the HC group, but some connections became disconnected or weaker in patients with PD. For instance, the connection between IC 51 and IC 54 was, directly and indirectly, positive in HCs (marked as H and H′ in the top panel), while this connection became completely disconnected in PD (marked as a, and a′ in the bottom panel). In addition, the direct connection between IC 54 and IC 33 was negative in HCs (marked as I in top panel), while this connection became positive in the PD patient group (marked as b in bottom panel). The changes in the connection strength resulted in new hieratical clustering in the PD patient group, and therefore, the RSNs were grouped in 3 clusters in PD. Alteration of the RSN clustering from HCs to PD patients is shown in Figure 6, in which HCs have a cluster corresponding to the DMN, and this cluster dissolved into three other clusters in PD patients.

## 4 DISCUSSION

In our study, we examined changes in functional brain connectivity architecture on a whole brain and network level in patients with PD. Previous studies have reported that the connectivity of brain regions at rest was affected by PD (Baggio, Segura, Sala‐Llonch, et al., 2015; Manes et al., 2018; Tessitore, Esposito, et al., 2012; Wu et al., 2009). In our study, various analyses were performed to investigate different aspects of the alteration of the whole‐brain RSNs. Large‐scale disruption of the brain network in PD was evaluated by the intraaspects and interaspects of RSN connectivity associated with PD. While the majority of previous studies have investigated the alteration of functional connectivity in PD by using seed‐based analysis, we employed a data‐driven approach based on group ICA to extract and evaluate RSNs.

We identified 23 RSNs in the PD patient and HC groups, which were composed of several subnetworks. Previous studies also extracted RSNs using group ICA (Onu et al., 2015; Yao et al., 2014). For example, a study based on group ICA of fMRI Toolbox (GIFT) extracted 40 ICs and characterized specific network topographies using rs‐fMRI data to differentiate PD and healthy subjects by logistic regression (Vo et al., 2017). Our results of group ICA and extracted RSNs (Figure 2) are in agreement with previous findings for patterns of spontaneous brain activities (Abou Elseoud et al., 2011; Foroutannia et al., 2020; Van Calster et al., 2017; Vergara et al., 2017).

Despite extracting group RSNs from the PD patient and HC groups, we introduced and measured the RSN scores. We observed that the RSN scores were significantly different in both groups (Figure 3). In line with previous resting‐state studies (Hacker et al., 2012), we found significantly reduced connectivity within 7 RSNs in PD patients compared with HCs. These RSNs were found in the lateral and medial posterior occipital cortices (IC 18), dorsal precentral gyri, central sulci, postcentral gyri, superior and inferior cerebellum (IC 19), ventral precentral gyri, central sulci, postcentral gyri, superior and inferior cerebellum (IC 38), limbic and medial temporal areas (IC 51), subgenual anterior cingulate cortex and orbitofrontal cortex (IC 54), and transverse temporal gyri (IC 60 and 38). The difference in mean RSN scores among PD patients and HCs was particularly striking on the lateral and medial posterior occipital cortices (IC 18) with *p* < .0052. Previous studies showed alteration of the visual pattern in PD, which may be associated with the mechanism of visual hallucinations in PD (Shine et al., 2015). The links between the visual changes and other features of patients with PD suggested visual dysfunction as a marker of dementia in these patients (Weil et al., 2016). Overall, other investigators have also found a widespread and significant reduction in connectivity in PD patients compared with HCs at rest in different brain areas (Dubbelink et al., 2014).

The RSN score used in the current study has a number of advantages. First, the RSN scores were extracted from the whole‐brain rs‐fMRI dataset using an ICA and dual‐regression method, without any a priori assumption on the pathophysiology of PD. This finding is in contrast to previous studies where parameters were extracted using predefined masks of regions. Second, this score can be used in statistical approaches and machine‐learning algorithms to distinguish patients with PD from other groups.

We used the ICA dual‐regression approach for extracting subject‐specific spatial RSNs and deriving the functional connectivity network of group RSNs. Once the RSNs were estimated, their spatial distributions were compared voxel‐wise among the two groups (Littow et al., 2010; Yu et al., 2015). The two regions with significantly different voxel‐wise connectivity in PD patients compared with HCs were the cerebellum and occipital pole, which are not part of RSNs (Figure 4). These results are in agreement with our RSN score analysis in that there was a significant decrease in this score in the superior and inferior cerebellum (IC 38) and lateral and the medial posterior occipital cortices (IC 18). The research of the abnormal cerebellar connectivity patterns in Freezing of Gait (FoG) and PD showed FC was lower in the prefrontal and parieto‐occipital cortices in PD‐FOG than in HC(Bharti et al., 2019). Moreover, other investigators have reported a reduction in the connectivity of the frontal and occipital regions (De Schipper et al., 2018; Göttlich et al., 2013) and spatial variations of the functional connectivity in Parkinsonism versus HC (Baggio, Segura, Garrido‐Millan, et al., 2015; Chen et al., 2015). The study suggested that altered connectivity of the cerebellum contributes to the pathophysiology of the cerebellar locomotor region, supporting the hypothesis that abnormal cerebellar function underlies motor function in PD.

We also considered both direct and indirect functional communication patterns between distinct RSNs. Based on dual‐regression analysis, we investigated the alteration of the brain network in PD compared with HCs and found disintegration and disruption of the RSNs, and in particular the DMN, in PD patients. Functional connectivity between RSNs in our analyses indicated four main clusters of RSNs in HCs (top panel in Figure 5) and three main clusters in patients with PD (bottom panel in Figure 5 down). Our results for healthy subjects are in agreement with previous studies that reported multiple clusters in the motor, executive, and visual networks in these subjects (Onu et al., 2015; Smith et al., 2013). As shown in Figure 6, the 4 clusters among HCs were reduced to 3 clusters in PD. The first cluster in HCs (purple lines) contains IC 12, 41, 4, 38, and 8, which were also seen in the first cluster of PD patients (purple lines). The second cluster in HCs (red lines) includes IC 14, 18, 30, 33, 42, 44, and 37, which were also seen in PD patients (red lines). This was also true for the fourth cluster among HCs (blue lines), which contains IC 19, 60, 96, 63, and 68. The only difference between the PD patient and HC groups was related to the third cluster, which contains IC 29, 76, 39, 54, and 51 in HCs. This cluster was related to the DMN (Figure 6). Our results for disruption of the DMN in PD is consistent with recent findings that the integrity of the DMN is decreased in the resting state and during the performance of executive tasks in PD (Ghahremani et al., 2018; Thilo van Eimeren et al., 2009). Seed‐based analyses also revealed reduced within‐DMN connectivity, a finding in line with a resting‐state fMRI study that evaluated cognitively unimpaired PD patients (Tessitore, Esposito, et al., 2012).

Study limitations: Several factors affected the results that we mentioned as the most important. First, it is important to say the fact that the limited number of data set may be affected by the result of the group rest network. Thus, we plan to carry out this research by larger developmental datasets. However, the main aim of this study was to investigate a comprehensive study on the group and PD resting‐state network. Another possible criticism regarding resting‐state network analysis is choosing and comparing with the template references of resting network and the limit number of predefined RSNs. However, in some regions, such as the cerebellum, we got more than one network.

## 5 CONCLUSION

In the current study, various analyses were performed to investigate different aspects of the alteration of the whole‐brain resting‐state networks. While the majority of the previous studies have investigated the alteration of the functional connectivity in PD using seed‐based analysis, we employed a data‐driven approach based on group ICA to extract and evaluate resting‐state networks. Based on dual‐regression analysis, changes in the network structure, as well as the connectivity network, were analyzed. Functional communication patterns between distinct RSNs were examined. We found disintegration and disruption of the DMN in PD.

## CONFLICT OF INTEREST

The authors declare that they have no conflict of interest.

## AUTHOR CONTRIBUTION

M. G. and A. F. have conceived the idea and done coding and engineering aspects. A. BF. has validated the result and analysis. M. G. and A. F. have written the paper, and A. BF. has edited it. M. G. has supervised the project and provided access to crucial research components.

## INFORMED CONSENT

Informed consent was obtained from all individual participants included in this study.

### PEER REVIEW

The peer review history for this article is available at https://publons.com/publon/10.1002/brb3.2101.

## Data Availability

This clinical study will be conducted according to the protocol and in compliance with Good Clinical Practice (GCP), with the Declaration of Helsinki1 (Version 2008) and with other applicable regulatory requirements. [Taken from PPMI website, document title: “Clinical Study Protocol”].
